# Naturally enhanced neutralizing breadth against SARS-CoV-2 one year after infection

**DOI:** 10.1038/s41586-021-03696-9

**Published:** 2021-06-14

**Authors:** Zijun Wang, Frauke Muecksch, Dennis Schaefer-Babajew, Shlomo Finkin, Charlotte Viant, Christian Gaebler, Hans- Heinrich Hoffmann, Christopher O. Barnes, Melissa Cipolla, Victor Ramos, Thiago Y. Oliveira, Alice Cho, Fabian Schmidt, Justin Da Silva, Eva Bednarski, Lauren Aguado, Jim Yee, Mridushi Daga, Martina Turroja, Katrina G. Millard, Mila Jankovic, Anna Gazumyan, Zhen Zhao, Charles M. Rice, Paul D. Bieniasz, Marina Caskey, Theodora Hatziioannou, Michel C. Nussenzweig

**Affiliations:** 1 Laboratory of Molecular Immunology, The Rockefeller University, New York, NY USA; 2 Laboratory of Retrovirology, The Rockefeller University, New York, NY USA; 3 Laboratory of Virology and Infectious Disease, The Rockefeller University, New York, NY USA; 4 Division of Biology and Biological Engineering, California Institute of Technology, Pasadena, CA USA; 5 Department of Pathology and Laboratory Medicine, Weill Cornell Medicine, New York, NY USA; 6 Howard Hughes Medical Institute, New York, NY USA

**Keywords:** Antibodies, Antimicrobial responses, SARS-CoV-2

## Abstract

More than one year after its inception, the coronavirus disease 2019 (COVID-19) pandemic caused by severe acute respiratory syndrome coronavirus 2 (SARS-CoV-2) remains difficult to control despite the availability of several working vaccines. Progress in controlling the pandemic is slowed by the emergence of variants that appear to be more transmissible and more resistant to antibodies^1,2^. Here we report on a cohort of 63 individuals who have recovered from COVID-19 assessed at 1.3, 6.2 and 12 months after SARS-CoV-2 infection, 41% of whom also received mRNA vaccines^3,4^. In the absence of vaccination, antibody reactivity to the receptor binding domain (RBD) of SARS-CoV-2, neutralizing activity and the number of RBD-specific memory B cells remain relatively stable between 6 and 12 months after infection. Vaccination increases all components of the humoral response and, as expected, results in serum neutralizing activities against variants of concern similar to or greater than the neutralizing activity against the original Wuhan Hu-1 strain achieved by vaccination of naive individuals^2,5–8^. The mechanism underlying these broad-based responses involves ongoing antibody somatic mutation, memory B cell clonal turnover and development of monoclonal antibodies that are exceptionally resistant to SARS-CoV-2 RBD mutations, including those found in the variants of concern^4,9^. In addition, B cell clones expressing broad and potent antibodies are selectively retained in the repertoire over time and expand markedly after vaccination. The data suggest that immunity in convalescent individuals will be very long lasting and that convalescent individuals who receive available mRNA vaccines will produce antibodies and memory B cells that should be protective against circulating SARS-CoV-2 variants.

## Abstract (web summary)

Antibodies against SARS-CoV-2 continue to evolve 6 to 12 months after infection in patients who have recovered from COVID-19, increasing in potency and breadth with time.

## Main

**Extended Data Fig. 1:**
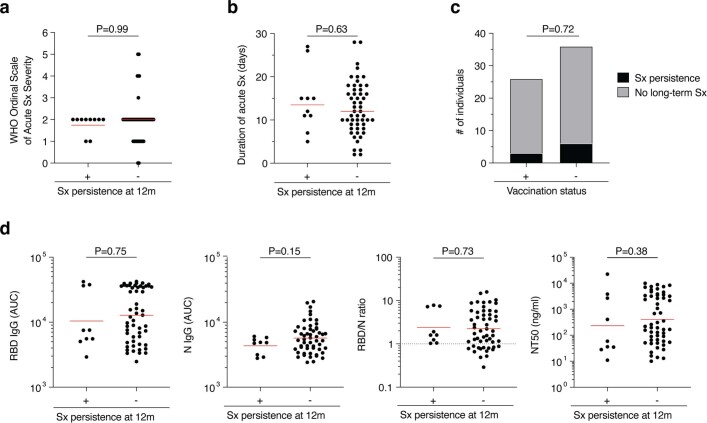
Clinical correlations. **a**–**d**, Association of persistence of symptoms (Sx) 12 months after infection with various clinical and serological parameters in our cohort of individuals who recovered from COVID-19 (*n* = 63). **a**, **b**, Acute disease severity as assessed with the WHO Ordinal Scale of Clinical Improvement (**a**, *P* = 0.99) and duration of acute phase symptoms (**b**, *P* = 0.63) in individuals reporting persistent symptoms (+) compared to individuals who are symptom-free (−) 12 months post-infection. **c**, Proportion of individuals reporting persistent symptoms (black area) compared to individuals who are symptom-free (grey area) 12 months after infection grouped by vaccination status (*P* = 0.72). **d**, Anti-RBD IgG (*P* = 0.75), anti-N IgG (*P* = 0.15), the RBD/N IgG ratio (*P* = 0.73), and NT_50_ titers (*P* = 0.38) at 12 months after infection in individuals reporting persistent symptoms (+) compared to individuals who are symptom-free (−) 12 months post-infection. Statistical significance was determined using the two-tailed Mann–Whitney test in **a**, **b**, **d**, and using the two-sided Fisher’s exact test in **c**.

We initially characterized immune responses to SARS-CoV-2 in a cohort of patients who have recovered from COVID-19 infection (hereafter referred to as convalescent individuals) 1.3 and 6.2 months after infection^3,4^. Between 8 February and 26 March 2021, 63 participants between the ages of 26 and 73 years old (median 47 years old) returned for a 12-month follow-up visit. Among those, 26 (41%) had received at least one dose of either the Moderna (mRNA-1273) or Pfizer-BioNTech (BNT162b2) vaccines, on average 40 days (range 2–82 days) before their study visit and 311 days (range 272–373 days) after the onset of acute illness (Supplementary Table 1). Participants were almost evenly split between the sexes (43% female) and of the individuals who returned for the 12-month follow-up, only 10% had been hospitalized and the remainder had experienced relatively mild initial infections. Only 14% of the individuals reported persistent long-term symptoms after 12 months, reduced from 44% at the 6-month time point^4^. Symptom persistence was not associated with the duration and severity of acute disease or with vaccination status (Extended Data Fig. 1a–c). All participants tested negative for active infection at the 12-month time point as measured by a saliva-based PCR assay^4^. The demographics and clinical characteristics of the participants are shown in Supplementary Tables 1, 2.

## Plasma SARS-CoV-2 antibody reactivity

**Fig. 1:**
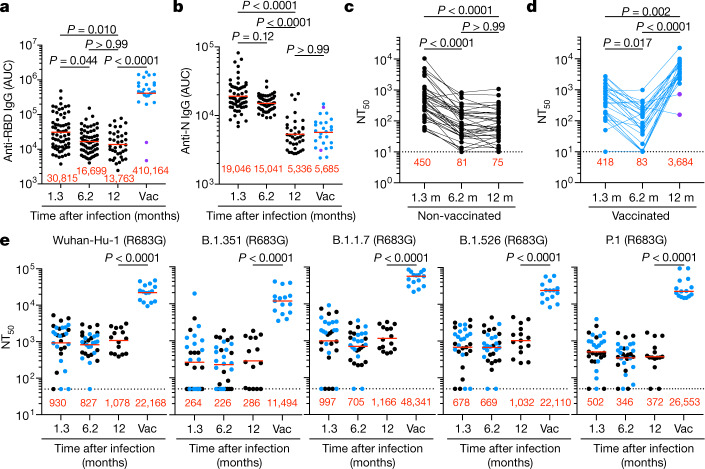
Plasma ELISAs and neutralizing activity. **a**–**d**, Plasma IgG antibody binding to SARS-CoV-2 RBD (**a**) and N protein (**b**) shown as area under the curve (AUC; numbers in red are mean geometric AUC), and plasma neutralizing activity (NT_50_) in unvaccinated (**c**) and vaccinated (vac) (**d**) individuals 12 months after SARS-CoV-2 infection (*n* = 63). *n* = 63 individuals, 37 convalescent unvaccinated (black) and 26 convalescent vaccinated (blue) individuals. **a**, **b**, Two-sided Kruskal–Wallis test with subsequent Dunn’s multiple comparisons. **c**, **d**, Lines connect longitudinal samples from the same individual. Two-sided Friedman test with subsequent Dunn’s multiple comparisons. Two individuals who received their first dose of vaccine 24–48 h before sample collection are represented in purple. **e**, Plasma neutralizing activity against indicated SARS-CoV-2 variants of concern (*n* = 30, 15 convalescent and 15 convalescent vaccinated individuals). The B.1.526 variant used here contains the E484K substitution. Substitutions, deletions and insertions in S variants used here are described in Methods. Two-tailed Mann–Whitney test. Red numbers in **c**–**e** indicate the geometric mean NT_50_ at the indicated time point. All experiments were performed at least in duplicate.

**Extended Data Fig. 2:**
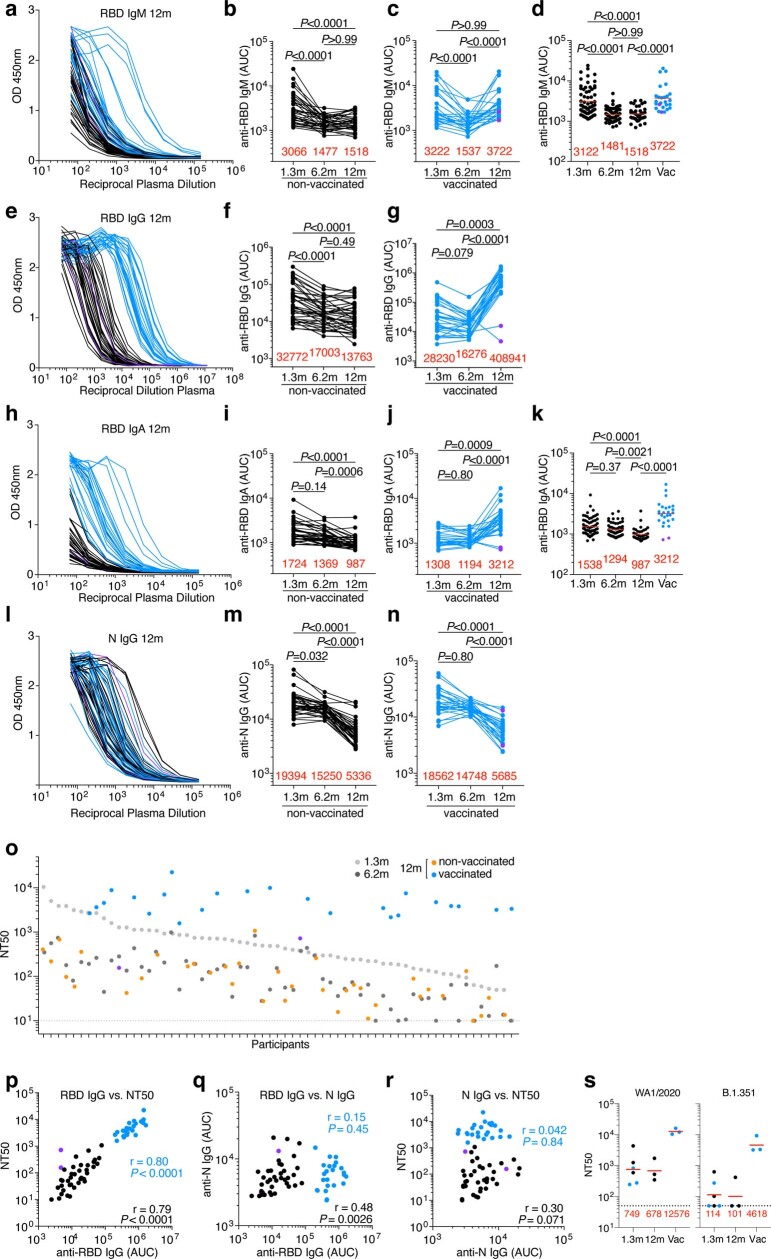
Plasma activity. **a**–**h**, ELISA results for plasma against SARS-CoV-2 RBD 12 months after infection (*n* = 63). Non-vaccinated individuals are depicted with black circles and lines, and vaccinated individuals are depicted in blue throughout. Two outlier individuals who received their first dose of vaccine 24–48 h before sample collection is depicted as purple circles. **a**–**n**, IgM (**a**–**d**) IgG (**e**–**g**) and IgA (**h**–**k**) antibody binding to SARS-CoV-2 RBD and IgG binding to N (**l**–**n**) 12 months after infection. **a**, **e**, **h**, **i**, ELISA curves from non-vaccinated (black lines) individuals, as well as individuals who received one or two doses (blue lines) of a COVID-19 mRNA vaccine (left panels). Area under the curve (AUC) over time in non-vaccinated (**b**, **f**, **i**, **m**) and vaccinated individuals (**c**, **g**, **j**, **n**). Lines connect longitudinal samples. **d**, **k**, Boxplots showing AUC values of all 63 individuals, as indicated. **o**, ranked average NT_50_ at 1.3 months (light grey) and 6.2 months (dark grey), as well as at 12 months for non-vaccinated (orange) individuals, and individuals who received one or two doses (blue circles) of a COVID-19 mRNA vaccine, respectively. Two individuals who received their first dose of vaccine 24–48 h before sample collection is depicted in purple. **p**–**r**, Correlation of serological parameters in non-vaccinated (black circles and black statistics) and vaccinated (blue circles and blue statistics) individuals. Two individuals who received their first dose of vaccine 24-48 h before sample collection is depicted as purple circles. Correlation of 12-month titers of anti- RBD IgG and NT_50_ (**p**), anti-RBD IgG and N IgG (**q**), and anti-N IgG and NT_50_ (**r**). **s**, Plasma neutralizing activity against authentic virus isolates WA1/2020 and B.1.351, as indicated (*n* = 6). Statistical significance was determined using two-sided Friedman test with subsequent Dunn’s multiple comparisons (**b**, **c**, **f**, **g**, **i**, **j**, **m**, **n**), or two-sided Kruskal–Wallis test with subsequent Dunn’s multiple comparisons (**d**, **k**) or using the Spearman correlation test for the non-vaccinated and vaccinated subgroups independently (**p**–**r**) or using two-tailed Mann–Whitney test (**s**). Red numbers indicate the geometric mean NT_50_ at the indicated time point. All experiments were performed at least in duplicate.

Antibody reactivity in plasma to the RBD and nucleoprotein (N) were measured by enzyme-linked immunosorbent assay (ELISA)^3^. We limited our analysis to RBD because plasma RBD antibodies are strongly correlated with neutralizing activity^3,10–12^. Convalescent participants who had not been vaccinated maintained most of their anti-RBD IgM (103%), IgG (82%) and IgA (72%) titres between 6 and 12 months after infection (Fig. 1a, Extended Data Fig. 2a–k). Consistent with previous reports^5–8^, vaccination increased the plasma RBD antibody levels, with IgG titres increasing by nearly 30-fold compared with unvaccinated individuals (Fig. 1a, right). The two individuals who did not show an increase in antibody titre had been vaccinated only two days before sample collection. In contrast to anti-RBD antibody titres that were relatively stable, anti-N antibody titres decreased significantly between 6 and 12 months in this assay, independently of vaccination status (Fig. 1b, Extended Data Fig. 2l–n).

Plasma neutralizing activity in 63 participants was measured using a human immunodeficiency virus 1 (HIV-1) pseudotyped with the SARS-CoV-2 spike (S) protein^3,4,13^ (Fig. 1c, d, Extended Data Fig. 2o). Twelve months after infection, the geometric mean half-maximal neutralizing titre (NT_50_) for the 37 individuals who had not been vaccinated was 75, which was not significantly different from the NT_50_ for the same individuals at 6.2 months after infection (Fig. 1c). By contrast, the vaccinated individuals showed a geometric mean NT_50_ of 3,684, which was nearly 50-fold higher than that of unvaccinated individuals and slightly higher than the 30-fold increase in anti-RBD IgG antibodies (Fig. 1a, c, d). Neutralizing activity was directly correlated with IgG anti-RBD (Extended Data Fig. 2p) but not with anti-N titres (Extended Data Fig. 2q, r). We conclude that neutralizing titres remain relatively unchanged between 6 and 12 months after SARS-CoV-2 infection, and that vaccination further boosts this activity by nearly 50-fold.

To determine the neutralizing activity against circulating variants of concern or interest, we performed neutralization assays on HIV-1 virus pseudotyped with the S protein of the following SARS-CoV-2 variants of concern or interest: B.1.1.7 (Alpha), B.1.351 (Beta), B.1.526 (Iota) and P.1 (Gamma)^1,14,15^. Twelve months after infection, neutralizing activity against the variants was generally lower than against wild-type SARS-CoV-2 virus in the same assay, with the greatest loss of activity against B.1.351 (Fig. 1e). After vaccination the geometric mean NT_50_ increased to 11,493, 48,341, 22,109 and 26,553 against B.1.351, B.1.1.7, B.1.526 and P.1, respectively. These titres are an order of magnitude higher than the neutralizing titres that have been reported against wild-type SARS-CoV-2 at the peak of the initial response in infected individuals and in naive individuals receiving both doses of mRNA vaccines^2–8^ (Fig. 1d). Similar results were also obtained using authentic SARS-CoV-2 WA1/2020 and B.1.351 (Extended Data Fig. 2s).

## Memory B cells

**Fig. 2:**
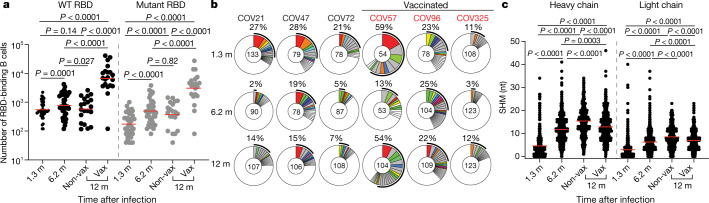
SARS-CoV-2 RBD-specific B cell memory. **a**, Number of antigen-binding memory B cells per 2 × 10^6^ B cells (Extended Data Fig. 5b, c) obtained at 1.3, 6.2 and 12 months after infection from 40 randomly selected individuals (vaccinated, *n* = 20; non-vaccinated, *n* = 20). Each dot represents one individual. Red horizontal bars indicate geometric mean values. Two-sided Kruskal–Wallis test with subsequent Dunn’s multiple comparisons. WT, wild type. **b**, The distribution of antibody sequences from 6 individuals 1.3 (top) or 6.2 (middle) or 12 (bottom) months after infection^3,4^. The number in the inner circle indicates the number of sequences analysed for the individual whose identifier is denoted above the circle. Pie slice size is proportional to the number of clonally related sequences. The outer black arc indicates the frequency of clonally expanded sequences detected in each participant. Coloured slices indicate persisting clones (same IGV and IGJ genes, with highly similar CDR3 sequences) found at both time points in the same participant. Grey slices indicate clones unique to the time point. White indicates sequences isolated once, and white slices indicate singlets found at both time points. **c**, Number of somatic nucleotide mutations (SHM) in the IGVH and IGVL genes (Supplementary Table 3) obtained after 1.3 or 6.2 or 12 months (1.3 month, *n* = 889; 6.2 month, *n* = 975; 12 month, *n* = 1,105 (unvaccinated, *n* = 417; vaccinated, *n* = 688)). Red horizontal bars indicate mean values. Two-sided Kruskal–Wallis test with subsequent Dunn’s multiple comparisons.

**Extended Data Fig. 3:**
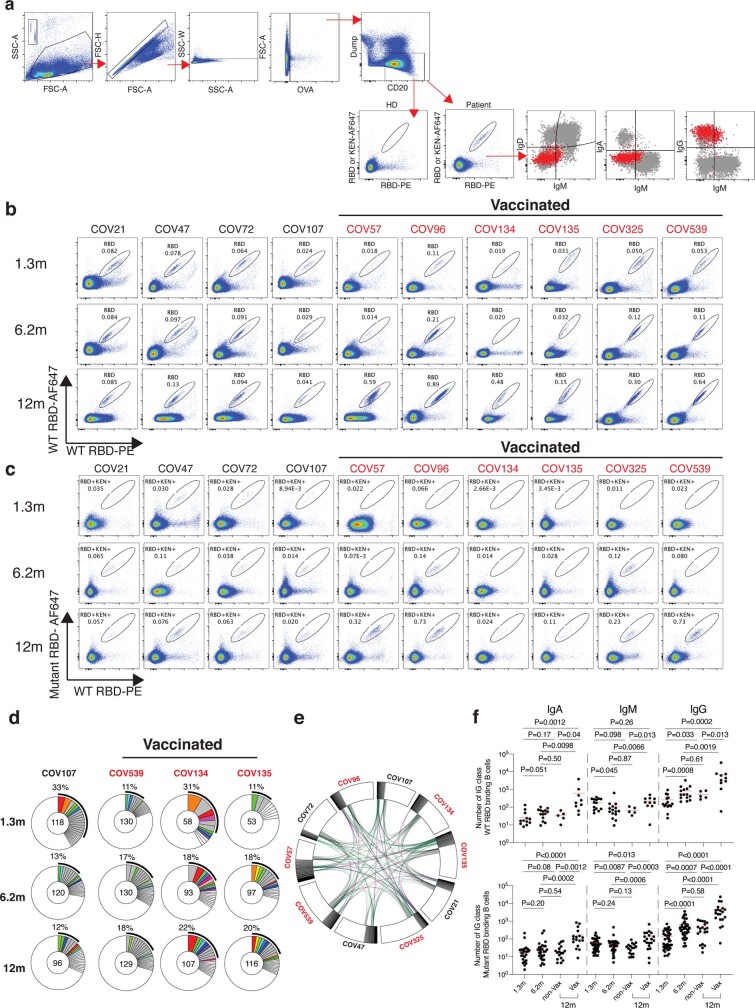
Flow cytometry. **a**, Gating strategy. Gating was on singlets that were CD20^+^ and CD3-CD8-CD16-Ova-. Anti-IgG, IgM, and IgA antibodies were used for B cell phenotype analysis. Sorted cells were RBD-PE^+^ and RBD/KEN-AF647^+^. **b**, **c**, Flow cytometry showing the percentage of RBD-double positive (**b**) and 647-K417N/E484K/N501Y mutant RBD cross-reactive (**c**) memory B cells from 1.3 or 6- and 12-months post-infection in 10 selected participants. **d**, As in Fig. 2b, Pie charts show the distribution of antibody sequences from 4 individuals after 1.3^3^ (upper panel) or 6.2^4^ months (middle panel) or 12 months (lower panel). **e**, Circos plot depicts the relationship between antibodies that share V and J gene segment sequences at both IGH and IGL. Purple, green, and grey lines connect related clones, clones and singles, and singles to each other, respectively. **f**, Graph summarizes cell number (indicated in **b**, **c**) (per 2 million B cells) of immunoglobulin class of antigens binding memory B cells in samples obtained at 1.3, 6.2 and 12 months. Each dot is one individual. (Vaccinees, *n* = 20, and non-vaccinees, *n* = 20). Red horizontal bars indicate mean values. Statistical significance was determined using two-sided Kruskal–Wallis test with subsequent Dunn’s multiple comparisons.

The memory B cell compartment serves as an immune reservoir containing a diverse collection of antibodies^16,17^. Although antibodies to the N-terminal domain and other parts of S can also be neutralizing, we limited our analysis to memory B cells that produce anti-RBD antibodies because they are the most numerous and potent^18,19^. To count RBD-specific memory B cells, we performed flow cytometry using biotin-labelled RBD^3^ (Fig. 2a, Extended Data Fig. 3a, b). Without vaccination, the number of RBD-specific memory B cells present 12 months after infection was 1.35-fold lower than the earlier 6.2-month time point (*P* = 0.027, Fig. 2a). By contrast, and consistent with previous reports^5,8,20^, individuals who recovered from COVID-19 and received mRNA vaccines showed an average increase of 8.6-fold in the number of circulating RBD-specific memory B cells (Fig. 2a). We also counted B cells expressing antibodies that bound to both wild-type and K417N/E484K/N501Y mutant RBDs using flow cytometry (Extended Data Fig. 3c). The number of B cells cross-reacting with variant RBD was directly proportional to and 1.6- to 3.2-fold lower than the number of B cells binding to wild-type RBD (Fig. 2a).

**Extended Data Fig. 4:**
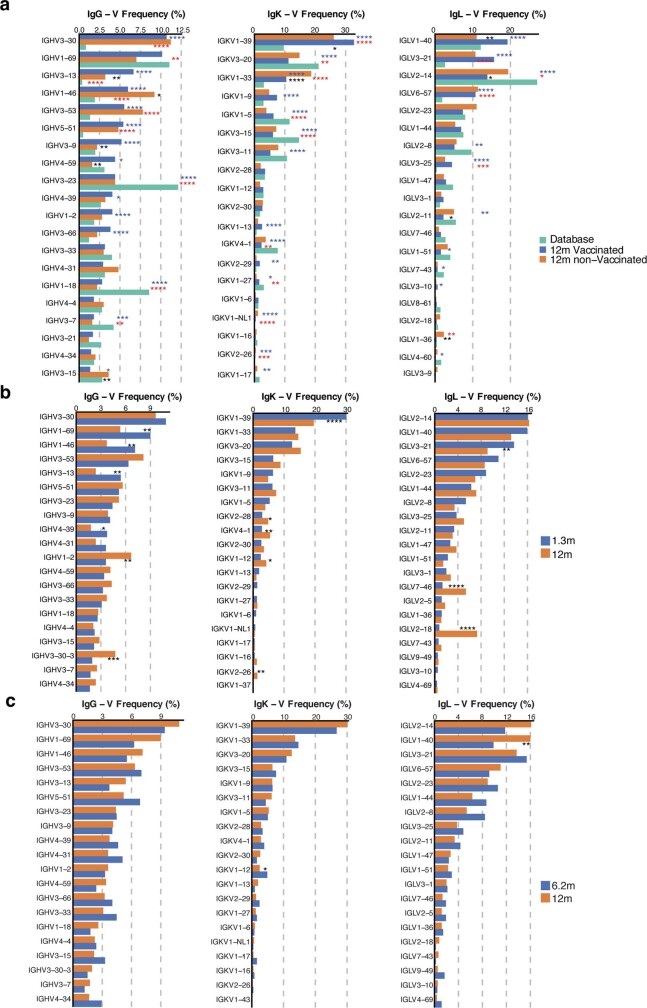
Frequency distribution of human V genes. Graph shows comparison of the frequency distributions of human V genes of anti-SARS-CoV-2 antibodies from donors at 1.3^3^, 6.2^4^, 12 months after infection. **a**, Graph shows relative abundance of human IGVH genes Sequence Read Archive accession SRP010970 (green), convalescent vaccinees (blue), and convalescent non-vaccinees (orange). Statistical significance was determined by two-sided binomial test. **b**, **c**, Same as in **a**, but showing comparison between antibodies from donors at 1.3 months^3^ (**b**), 6.2 month^4^ (**c**) and 12 months after infection. Two-sided binomial tests with unequal variance were used to compare the frequency distributions., significant differences are denoted with stars (* *P* < 0.05, ** *P* < 0.01, *** *P* < 0.001, **** = *P* < 0.0001).

The memory B cell compartment accumulates mutations and undergoes clonal evolution over the initial six months after infection^4,9,21,22^. To determine whether the memory compartment continues to evolve between 6 and 12 months after infection, we obtained 1,105 paired antibody heavy- and light-chain sequences from 10 individuals who were also assessed at the earlier time points, 6 of whom were vaccinated (Fig. 2b, Extended Data Fig. 3d, Supplementary Table 3). There were few significant differences among the expressed IGHV and IGLV genes between vaccinated and un-vaccinated groups, or between the 1.3-, 6-month and 1-year time points^3,4^ (Extended Data Fig. 4a–c). *IGHV3-30* and *IGHV3-53* remained over-represented independently of vaccination status^10,18^ (Extended Data Fig. 4a).

**Extended Data Fig. 5:**
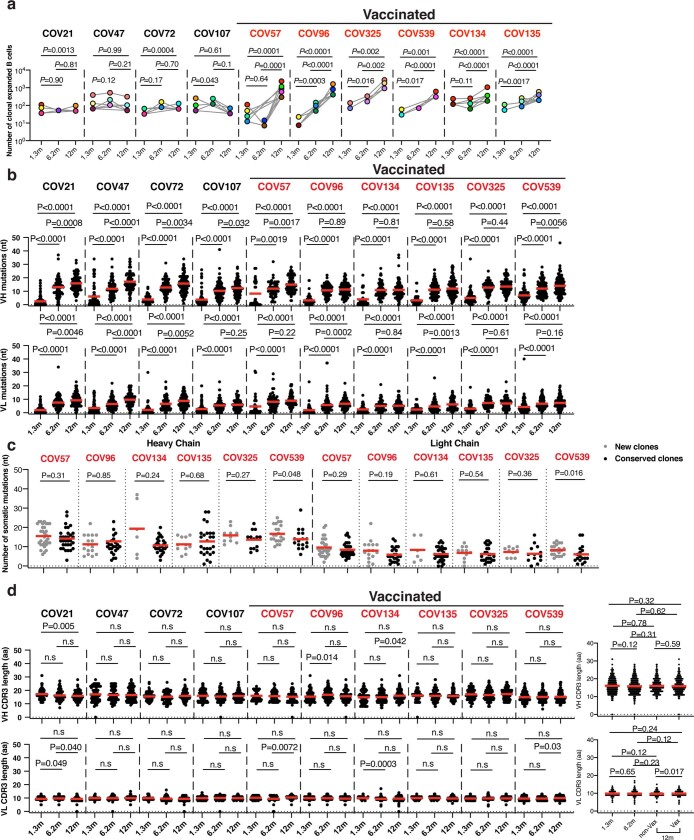
Analysis of anti-RBD antibodies. **a**, Number of clonally expanded B cells (per 10 million B cells) at indicated time points in 10 individuals. Colours indicate shared clones appearing at different time points. Statistical significance was determined using two-tailed Wilcoxon matched-pairs signed rank test. Vaccinees are marked in red. Statistical significance was determined using Wilcoxon matched-pairs signed rank tests. Vaccinees are marked in red. **b**, Number of somatic nucleotide mutations in the IGVH (top) and IGVL (bottom) in antibodies obtained after 1.3 or 6.2 or 12 months from the indicated individual. **c**, Same as **b**, but graphs show comparison between new clones and conserved clones in 6 vaccinated convalescent individuals at 12 months after infection. **d**, The amino acid length of the CDR3 s at the IGVH and IGVL for each individual. Right panel shows all antibodies combined. (1.3m: *n* = 889; 6.2m: *n* = 975; 12m: *n* = 1105, (non-vax: *n* = 417; vax: *n* = 688)). The horizontal bars indicate the mean. Statistical significance was determined using two-sided Kruskal–Wallis test with subsequent Dunn’s multiple comparisons (**a**, **b**, **d**), or two-tailed Mann–Whitney U-tests (**c**).

All individuals assayed at 12 months showed expansion of RBD-binding memory cell clones that expressed closely related IGHV and IGLV genes (Fig. 2b, Extended Data Fig. 3d, e). The relative fraction of cells belonging to these clones varied from 7% to 54% of the repertoire, with no significant difference between vaccinated and non-vaccinated groups. The overall clonal composition differed between 6 and 12 months after infection in all individuals, suggesting ongoing clonal evolution (Fig. 2b, Extended Data Fig. 3d). Among the 89 clones found 12 months after infection, 61% were not previously detected and 39% were present at one of the earlier time points (Fig. 2b, Extended Data Fig. 3d). In vaccinated individuals, the increase in size of the memory compartment was paralleled by an increase in the absolute number of B cells representing all persistent clones (Extended Data Fig. 5a). Thus, RBD-specific memory B cell clones were re-expanded upon vaccination in all six convalescent individuals examined (Fig. 2b, Extended Data Figs. 3d, 5a).

**Extended Data Fig. 6:**
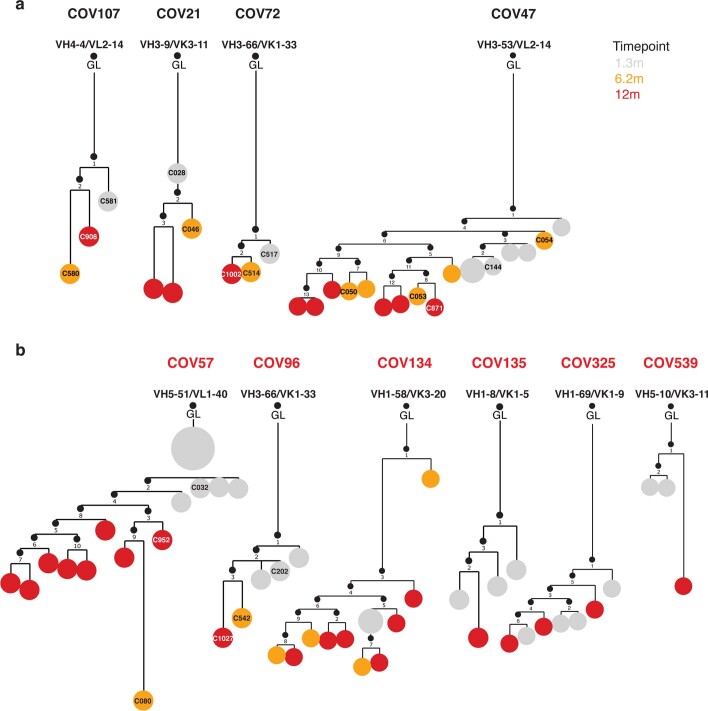
Evolution of anti-SARS-CoV-2 RBD antibody clone. Clonal evolution of RBD-binding memory B cells from ten convalescent individuals, **a**, Phylogenetic tree graph shows clones from convalescent non-vaccinees, **b**, Same as **a**, but from convalescent vaccinees. Numbers refer to mutations compared to the preceding vertical node. Colours indicate time point; grey, orange and red represent 1.3, 6 and 12 months respectively, black dots indicate inferred nodes, and size is proportional to sequence copy number; GL = germline sequence.

Somatic hypermutation of antibody genes continued between 6 and 12 months after infection (Fig. 2c). Slightly higher levels of antibody-gene mutation were found in individuals who had not been vaccinated compared with vaccinated individuals, possibly owing to recruitment of newly formed memory cells into the expanded memory compartment of the vaccinated individuals (Fig. 2c, Extended Data Fig. 5b). There was no significant difference in numbers of mutations between conserved and newly arising clones at the 12-month time point in vaccinated individuals (Extended Data Fig. 5c). Moreover, phylogenetic analysis revealed that sequences found at 6 and 12 months after infection were intermingled and similarly distant from their unmutated common ancestors (Extended Data Fig. 6). We conclude that clonal re-expansion of memory cells in response to vaccination is not associated with additional accumulation of large numbers of somatic mutations as might be expected if the clones were re-entering and proliferating in germinal centres.

## Neutralizing activity of monoclonal antibodies

**Fig. 3:**
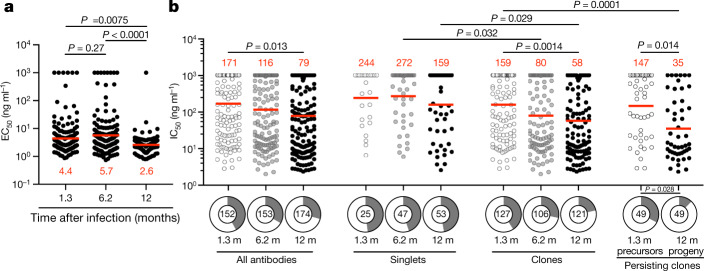
Anti-SARS-CoV-2 RBD monoclonal antibodies. **a**, EC_50_ for SARS-CoV-2 RBD of antibodies isolated at 1.3 (*n* = 152) 6.2 (*n* = 153) and 12 (*n* = 174) months after infection^3,4^, determined by ELISA. Two-sided Kruskal–Wallis test with subsequent Dunn’s multiple comparisons (1.3 months versus 6.2 months, *P* = 0.27; 1.3 months versus 12 months, *P* = 0.0075; 6.2 versus 12 months, *P* < 0.0001). **b**, SARS-CoV-2-neutralizing activity of monoclonal antibodies measured using a SARS-CoV-2 pseudovirus neutralization assay^3,13^. IC_50_ values for antibodies isolated at 1.3, 6.2 and 12 months after infection against wild-type SARS-CoV-2 (Wuhan-Hu-1 strain^41^) are shown. Each dot represents one antibody. Pie charts illustrate the fraction of non-neutralizing (IC_50_ > 1,000 ng ml^−1^) antibodies (grey slices), inner circle shows the number of antibodies tested per group. Horizontal bars and red numbers indicate geometric mean values. Statistical significance was determined through the two-sided Kruskal–Wallis test with subsequent Dunn’s multiple comparisons.

**Extended Data Fig. 7:**
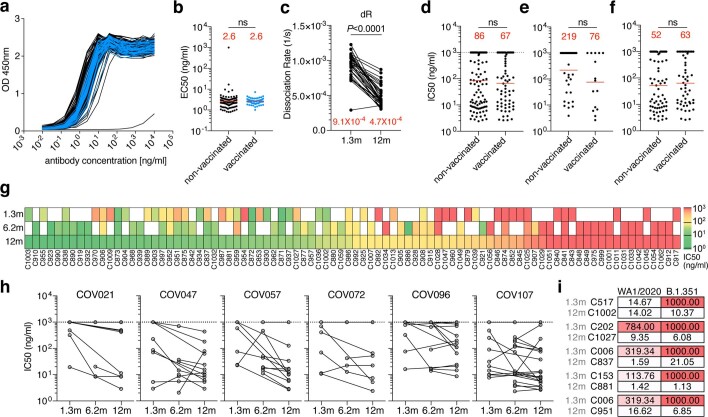
WT RBD binding and pseudovirus neutralization. **a**, **b**, Binding curves (**a**) and EC_50_ dot plot (**b**) of mAbs isolated from non-vaccinated (black curves and dots) and from vaccinated (blue curves and dots) convalescents individuals 12 months after infection (*P* = 0.74). **c**, Avidity (dissociation rate) measuring plasma reactivity to RBD at the 1.3- and 12 month follow-up visit (*n* = 33). **d**–**f**, IC_50_ values of mAbs isolated 12 months after infection from non-vaccinated and vaccinated individuals; all 12 month antibodies irrespective of clonality (**d**), singlets only (**e**), and only antibodies belonging to a clone or shared over time (**f**). Statistical significance in **b**, **d**–**f** was determined using the two-tailed Mann–Whitney test; two-tailed Wilcoxon test (**c**). The geometric mean EC_50_ and IC_50_ are indicated in red. **g**, Heat map shows the neutralizing activity of clonally related antibodies against wt-SARS-CoV-2 over time. White tiles indicate no clonal relative at the respective time point. Clones are ranked from left to right by the potency of the 12 month progeny antibodies which are denoted below the tiles. **h**, IC_50_ values of shared clones of mAbs cloned from B cells from the initial 1.3- and 6.2-, as well as 12 month follow-up visit, divided by participant, as indicated. Lines connect clonal antibodies shared between time points. Antibodies with IC_50_ > 1,000 ng/ml are plotted at 1,000 ng/ml in **d**–**h**. **i**, IC_50_ values of 5 neutralizing antibody pairs against indicated authentic SARS-CoV-2 WA1/2020 and B.1.351 viruses (*n* = 10). Average EC_50_ and IC_50_ values of two independent experiments are shown.

To determine whether the antibodies obtained from memory B cells 12 months after infection bind to RBD, we performed ELISAs (Fig. 3a). We tested 174 antibodies, including: (1) 53 that were randomly selected from those that appeared only once and only after 1 year; (2) 91 that appeared as expanded clones or singlets at more than one time point; and (3) 30 representatives of newly arising expanded clones (Supplementary Tables 4, 5). Among the 174 antibodies tested, 173 bound to RBD, indicating that the flow cytometry method used to identify B cells expressing anti-RBD antibodies was efficient (Supplementary Tables 4, 5). The geometric mean ELISA half-maximal concentration (EC_50_) of the antibodies obtained 12 months after infection was 2.6 ng ml^−1^, which was significantly lower than after 6 months, independently of vaccination status and suggestive of an increase in affinity (Fig. 3a, Extended Data Fig. 7a, b, Supplementary Tables 4, 5). Consistent with this observation, there was an overall increase in the apparent avidity of plasma antibodies between 1.3 and 12 months^3,4^ (*P* < 0.0001) (Extended Data Fig. 7c).

All 174 RBD binding antibodies obtained from the 12-month time point were tested for neutralizing activity in a SARS-CoV-2 pseudotype-neutralization assay. When compared with the earlier time points from the same individuals, the geometric mean half-maximal inhibitory concentration (IC_50_) improved from 171 ng ml^−1^ (at 1.3 months) to 116 ng ml^−1^ (at 6 months) to 79 ng ml^−1^ (at 12 months), with no significant difference between vaccinated and non-vaccinated individuals (Fig. 3b, Extended Data Fig. 7d, Supplementary Table 4). This increased potency was most evident in the antibodies expressed by expanded clones of B cells that were conserved for the entire observation period independently of vaccination status (*P* = 0.014) (Fig. 3b, right, Extended Data Fig. 7e–h, Supplementary Table 5). The overall increase in neutralizing activity among conserved clones was owing to accumulation of clones expressing antibodies with potent neutralizing activity and simultaneous loss of clones expressing antibodies with no measurable activity (*P* = 0.028) (Fig. 3b, bottom right). Consistent with this observation, antibodies obtained from clonally expanded B cells 12 months after infection were more potent than antibodies obtained from unique B cells at the same time point (*P* = 0.029) (Fig. 3b).

## Epitopes and breadth of neutralization

**Fig. 4:**
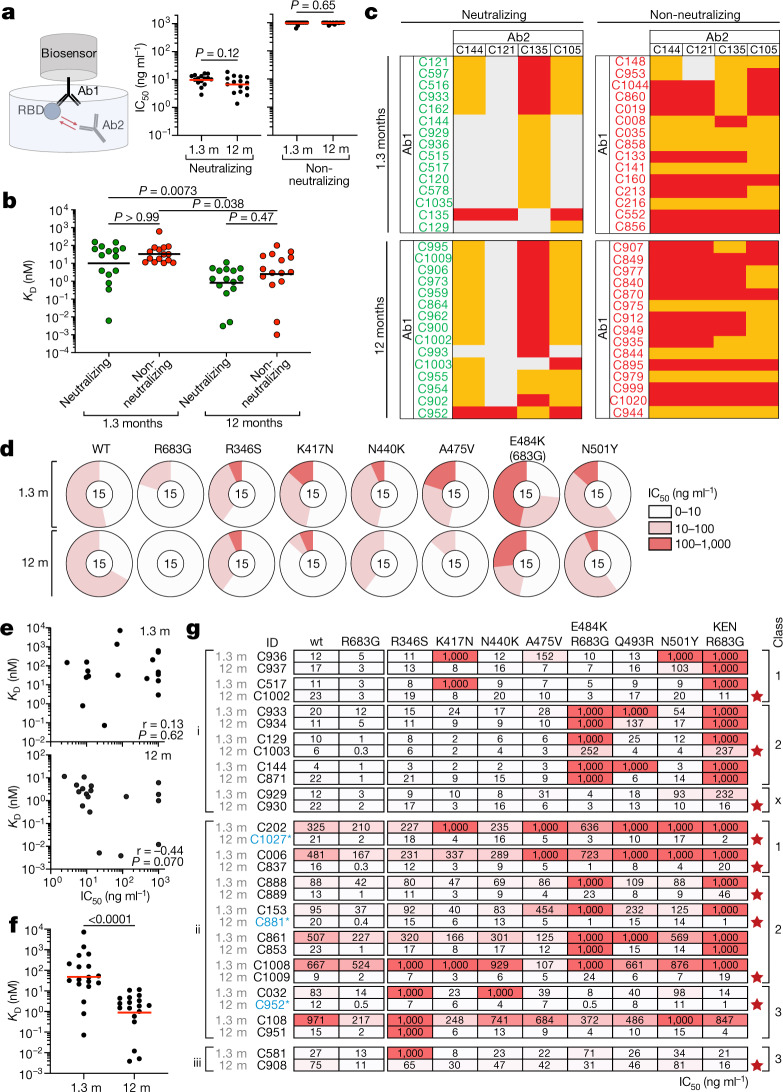
Epitope targeting and evolution of anti-SARS-CoV-2 RBD antibodies. **a**, Schematic of the BLI experiment (left) and IC_50_ values for randomly selected neutralizing and non-neutralizing antibodies (Ab1 and Ab2) isolated at 1.3 and 12 months after infection (*n* = 15 antibodies per group, *n* = 60 antibodies in total). Red horizontal bars indicate geometric mean. Two-sided Mann–Whitney test. **b**, Dissociation constants (*K*_D_) of the *n* = 30 neutralizing (green) and *n* = 30 non-neutralizing (red) antibodies shown in **a**. Horizontal bars indicate geometric mean values. Two-sided Kruskal–Wallis test with subsequent Dunn’s multiple comparisons. BLI traces are shown in Extended Data Fig. 8. **c**, Heat map of relative inhibition of binding of a monoclonal antibody (Ab2) to preformed complexes of RBD with another monoclonal antibody (Ab1) (grey, no binding; orange, intermediate binding; red, high binding). Data are normalized by subtraction of the autologous antibody control. BLI traces are shown in Extended Data Fig. 9. **d**, Neutralization of the indicated mutant RBD proteins with antibodies shown in **a**–**c**. Pie charts illustrate the fraction of antibodies that are poorly or non-neutralizing (IC_50_ of 100–1,000 ng ml^−1^, red), intermediate neutralizing (IC_50_ of 10–100 ng ml^−1^, pink) and potently neutralizing (IC_50_ of 0–10 ng ml^−1^, white) for each mutant. The number in the inner circle shows the number of antibodies tested. **e**, Graphs show affinities (*y*-axis) plotted against neutralization activity (*x*-axis) for 18 clonal antibody pairs isolated 1.3 (top) and 12 months (bottom) after infection (*n* = 36 antibodies). Spearman correlation test. **f**, BLI affinity measurements for same *n* = 36 paired antibodies as in **e**. Two-tailed Wilcoxon test. **g**, IC_50_ values for *n* = 30 paired neutralizing antibodies isolated at indicated time points versus indicated mutant SARS-CoV-2 pseudoviruses. Antibodies are divided into groups I, II and III (left), on the basis of neutralizing activity: I, potent clonal pairs that do not improve over time; II, clonal pairs that show increased activity over time; and III, clonal pairs showing decreased neutralization activity after 12 months. Antibody class assignment based on initial (1.3 month after infection) sensitivity to mutation is indicated on the right. Red stars indicate antibodies that neutralize all tested RBD mutants. Colour gradient indicates IC_50_ values ranging from 0 (white) to 1,000 ng ml^−1^ (red).

**Extended Data Fig. 8:**
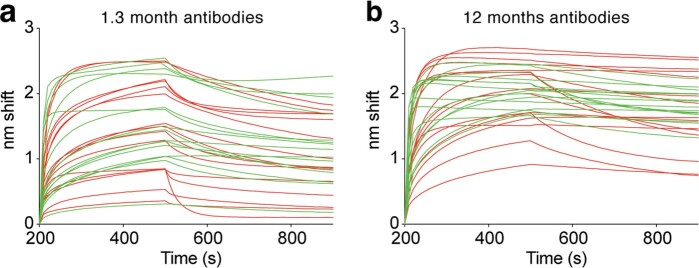
Biolayer interferometry affinity measurements. **a**, **b**, Graphs depict affinity measurements of neutralizing (green) and non-neutralizing (red) antibodies isolated 1.3 months (**a)** or 12 months (**b**) after infection.

**Extended Data Fig. 9:**
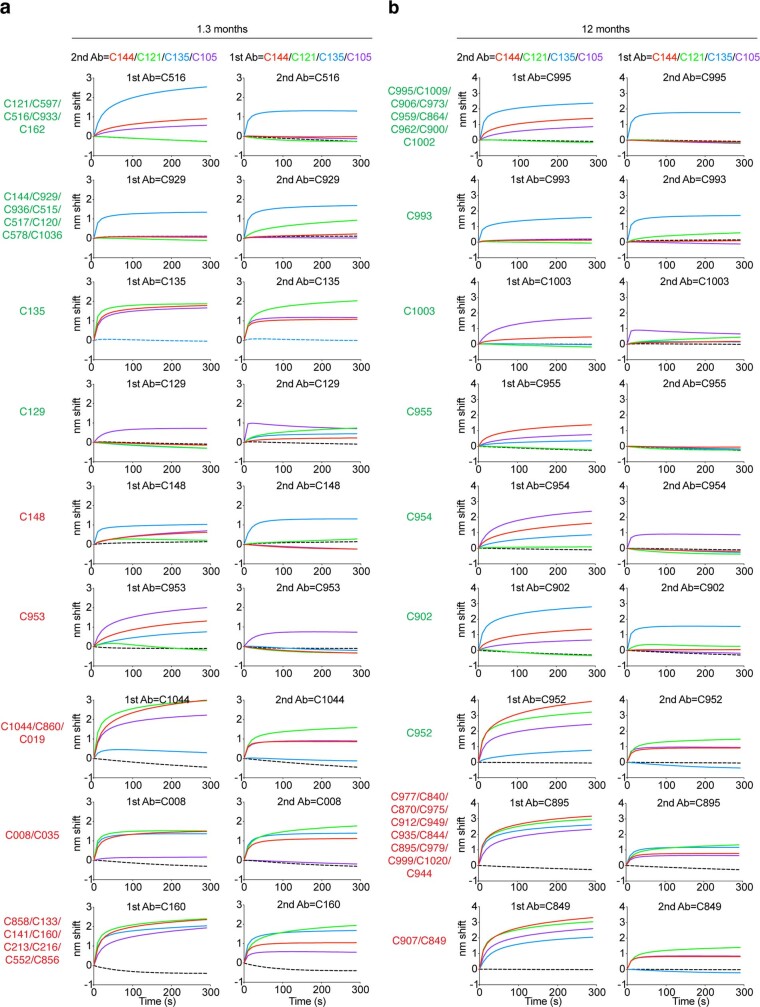
Biolayer interferometry antibody competition experiment. **a**, **b**, Anti-SARS-CoV-2 RBD antibodies isolated 1.3 (**a**) or 12 months (**b**) after infection were assayed for competition with structurally characterized anti-RBD antibodies by biolayer interferometry experiments as in Fig. 4a. Graphs represent the binding of the second antibody (2nd Ab) to preformed first antibody (1st Ab)–RBD complexes. Dotted line denotes when 1st Ab and 2nd Ab are the same. For each antibody group identified in Fig. 4c the left graphs represent the binding of the class-representative C144, C121, C135 or C105^3,23^ (2nd Ab) to the candidate antibody (1st Ab)-RBD complex. The right graphs represent the binding of the candidate antibody (2nd Ab) to the complex of C144-RBD, C121-RBD, C135-RBD or C105-RBD (1st Ab). Antibodies belonging to the same groups are indicated to the left of the respective curves.

To determine whether the loss of non-neutralizing antibodies over time was due to preferential loss of antibodies targeting specific epitopes, we performed biolayer interferometry (BLI) experiments in which a preformed antibody–RBD complex was exposed to a second monoclonal antibody targeting one of three classes of structurally defined epitopes^3,23^ (schematic in Fig. 4a). We assayed 60 randomly selected antibodies with comparable neutralizing activity from the 1.3- and 12-month time points. The 60 antibodies were evenly distributed between the two time points and between neutralizers and non-neutralizers (Fig. 4). Antibody affinities for RBD were similar among neutralizers and non-neutralizers obtained at the same time point (Fig. 4b, Extended Data Fig. 8). When the two sets of unrelated antibodies obtained 1.3 and 12 months after infection were compared, they showed increasing affinity over time independent of their neutralizing activity (Fig. 4b, Extended Data Fig. 8). In competition experiments, 2 out of 30 non-neutralizing antibodies inhibited binding of the class 1 (C105), 2 (C121 and C144) or 3 (C135) antibodies tested; the remaining 28 non-neutralizing antibodies must therefore bind to epitopes that do not overlap with the epitopes of these classes of antibodies (Fig. 4c, Extended Data Fig. 9). By contrast, 28 out of 30 neutralizing antibodies blocked class 1 or 2 antibodies whose target epitopes are structural components of the RBD that interact with its cellular receptor, angiotensin-converting enzyme 2 (ACE2)^23,24^ (Fig. 4c, Extended Data Fig. 9). In addition, whereas 9 of the 15 neutralizing antibodies obtained after 1.3 months blocked both class 1 and 2 antibodies, only 1 of the 15 obtained after 12 months did so. In contrast to the earlier time point, 13 of 15 neutralizing antibodies obtained after 12 months interfered only with C121, a class 2 antibody^3,23^ (Fig. 4c, Extended Data Fig. 9). We conclude that neutralizing antibodies are retained and non-neutralizing antibodies targeting RBD surfaces that do not interact with ACE2 are removed from the repertoire over time.

To determine whether there was an increase in neutralization breadth over time, the neutralizing activity of the 60 antibodies was assayed against a panel of RBD mutants covering residues associated with circulating variants of concern: R346S, K417N, N440K, A475V, E484K and N501Y (Fig. 4d, Supplementary Table 6). Increased activity was evident against K417N, N440K, A475V, E484K and N501Y (Fig. 4d, Supplementary Table 6). These data indicate that evolution of the antibody repertoire results in acquisition of neutralization breadth over time.

The increase in breadth and overall potency of memory B cell antibodies could be owing to shifts in the repertoire, clonal evolution or both. To determine whether changes in specific clones are associated with increases in affinity and breadth, we measured the relative affinity and neutralizing breadth of matched pairs of antibodies expressed by expanded clones of B cells that were maintained in the repertoire over the entire observation period^3,4^. SARS-CoV-2-neutralizing activity of the antibodies present at 1.3 or 12 months was not significantly correlated with affinity at either time point when each time point was considered independently (Fig. 4e). However, there was a significant increase in overall affinity over time, including in the 4 pairs of antibodies with no measurable neutralizing activity (Fig. 4f and Supplementary Table 7). Neutralizing breadth was assayed for 15 randomly selected pairs of antibodies targeting epitopes assigned to the 3 dominant classes of neutralizing antibodies^3,23,25,26^. Seven of the selected antibodies showed equivalent or decreased activity against wild-type SARS-CoV-2 after 12 months (Fig. 4g, Supplementary Table 8). However, neutralizing breadth increased between 1.3 and 12 months for all 15 pairs, even when neutralizing activity against the wild-type was unchanged or decreased (Fig. 4g, Supplementary Table 8). Only 1 out of the 15 antibodies obtained after 1.3 months neutralized all the mutants tested (Fig. 4g). By contrast, 10 out of the 15 antibodies obtained from the same clones 12 months after infection neutralized all variants tested, with IC_50_ values as low as 1 ng ml^−1^ against the RBD triple mutant K417N/E484K/N501Y found in B.1.351 (Fig. 4g, Supplementary Table 8). Similar results were obtained with authentic WA1/2020 and B.1.351 (Extended Data Fig. 7i). In conclusion, continued clonal evolution of anti-SARS-CoV-2 antibodies over 12 months favours increasing potency and breadth, resulting in monoclonal antibodies with exceptional activity against a wide group of variants.

## Discussion

During immune responses, activated B cells interact with cognate T cells and begin dividing before selection into the plasma cell, memory or germinal centre B cell compartments, partly on the basis of their affinity for antigen^17,27–31^. Whereas B cells expressing high-affinity antibodies are favoured to enter the long-lived plasma cell compartment, the memory compartment is more diverse and can develop directly from activated B cells or from a germinal centre^17,27–31^. Memory cells emanating from a germinal centre carry more mutations than those that develop directly from activated B cells because they undergo additional cycles of division^32^.

Consistent with the longevity of bone marrow plasma cells, infection with SARS-CoV-2 leads to persistent anti-RBD antibodies in serum, and corresponding neutralizing responses. Nearly 93% of the plasma neutralizing activity is retained between 6 and 12 months after infection^33,34^. Vaccination boosts the neutralizing response by 1.5 orders of magnitude by inducing additional plasma cell differentiation from the memory B cell compartment^5,7,35^. Recruitment of evolved memory B cells producing antibodies with broad and potent neutralizing activity into the plasma cell compartment is likely to account for the high serologic activity of vaccinated convalescent individuals against variants of concern^20,35,36^.

Less is known about selection and maintenance of the memory B cell compartment. SARS-CoV-2 infection produces a memory compartment that continues to evolve more than 12 months after infection with accumulation of somatic mutations, emergence of new clones and increasing affinity, all of which are consistent with long-term persistence of germinal centres. The increase in activity against SARS-CoV-2 mutants parallels the increase in affinity and is consistent with the finding that increasing the apparent affinity of anti-SARS-CoV-2 antibodies by dimerization or by creating bi-specific antibodies also increases resistance to RBD mutations^37–40^.

Continued antibody evolution in germinal centres requires antigen, which can be retained in these structures over long periods of time^29^. In addition, SARS-CoV-2 protein and nucleic acids have been reported to remain in the gut for at least two months after infection^4^. Regardless of the source of the antigen, antibody evolution favours epitopes overlapping with the ACE2-binding site on the RBD, possibly because these are epitopes that are preferentially exposed on trimeric S protein or virus particles.

Vaccination after SARS-CoV-2 infection increases the number of RBD-binding memory cells by more than an order of magnitude by recruiting new B cell clones into memory and expanding persistent clones. The persistent clones expand without accumulating large numbers of additional mutations, indicating that clonal expansion of human memory B cells does not require re-entry into germinal centres and occurs in the activated B cell compartment^17,27–31^.

The notable evolution of neutralizing breadth after infection with SARS-CoV-2 and the robust enhancement of serologic responses and B cell memory achieved with mRNA vaccination suggests that convalescent individuals who are vaccinated should enjoy high levels of protection against emerging variants without a need to modify existing vaccines. If memory responses evolve in a similar manner in naive individuals who receive vaccines, additional appropriately timed boosting with available vaccines should lead to protective immunity against circulating variants.

## Methods

### Data reporting

No statistical methods were used to predetermine sample size. The experiments were not randomized and the investigators were not blinded to allocation during experiments and outcome assessment.

### Study participants

Previously enrolled study participants were asked to return for a 12-month follow-up visit at the Rockefeller University Hospital in New York between 8 February and 26 March 2021. Eligible participants were adults with a history of participation in both prior study visits of our longitudinal cohort study of COVID-19 recovered individuals^3,4^. All participants had a confirmed history of SARS-CoV-2 infection, either diagnosed during the acute infection by PCR with reverse transcription (RT–PCR) or retrospectively confirmed by seroconversion. Exclusion criteria included presence of symptoms suggestive of active SARS-CoV-2 infection. Most study participants were residents of the Greater New York City tri-state region and were asked to return approximately 12 months after the time of onset of COVID-19 symptoms. Participants presented to the Rockefeller University Hospital for blood sample collection and were asked about potential symptom persistence since their 6.2-month study visit, laboratory-confirmed episodes of reinfection with SARS-CoV-2, and whether they had received any COVID-19-related treatment or SARS-CoV-2 vaccination in the interim. Study participants who had received COVID-19 vaccinations, were exclusively recipients of one of the two currently EUA-approved mRNA vaccines, Moderna (mRNA-1273) or Pfizer-BioNTech (BNT162b2), and individuals who received both doses did so according to current interval guidelines, namely 28 days (range 28–30 days) for Moderna and 21 days (range 21–23 days) for Pfizer-BioNtech. Detailed characteristics of the symptomology and severity of the acute infection, symptom kinetics, and the immediate convalescent phase (7 weeks post-symptom onset until 6.2-month visit) have previously been reported^4^. Participants who presented with persistent symptoms attributable to COVID-19 were identified on the basis of chronic shortness of breath or fatigue, deficit in athletic ability and/or three or more additional long-term symptoms such as persistent unexplained fevers, chest pain, new-onset cardiac sequalae, arthralgias, impairment of concentration/mental acuity, impairment of sense of smell/taste, neuropathy or cutaneous findings as previously described^4^. Clinical data collection and management were carried out using the software iRIS by iMedRIS. All participants at Rockefeller University provided written informed consent before participation in the study and the study was conducted in accordance with Good Clinical Practice. For detailed participant characteristics see Supplementary Table 2. The study was performed in compliance with all relevant ethical regulations and the protocol (DRO-1006) for studies with human participants was approved by the Institutional Review Board of the Rockefeller University.

### SARS-CoV-2 molecular tests

Saliva was collected into guanidine thiocyanate buffer as previously described^42^. RNA was extracted using either a column-based (Qiagen QIAmp DSP Viral RNA Mini Kit, catalogue (cat.) no. 61904) or a magnetic bead-based method as previously described^43^. Reverse-transcribed cDNA was amplified using primers and probes validated by the CDC or by Columbia University Personalized Medicine Genomics Laboratory, respectively and approved by the FDA under the Emergency Use Authorization. Viral RNA was considered detected if *C*_t_ for two viral primers/probes were <40.

### Blood samples processing and storage

Peripheral blood mononuclear cells obtained from samples collected at Rockefeller University were purified as previously reported by gradient centrifugation and stored in liquid nitrogen in the presence of FCS and DMSO^3,4^. Heparinized plasma and serum samples were aliquoted and stored at −20 °C or less. Prior to experiments, aliquots of plasma samples were heat-inactivated (56 °C for 1 h) and then stored at 4 °C.

### ELISAs

ELISAs^44,45^ to evaluate antibodies binding to SARS-CoV-2 RBD and N were performed by coating of high-binding 96-half-well plates (Corning 3690) with 50 μl per well of a 1 μg ml^−1^ protein solution in PBS overnight at 4 °C. Plates were washed 6 times with washing buffer (1× PBS with 0.05% Tween-20 (Sigma-Aldrich)) and incubated with 170 μl per well blocking buffer (1× PBS with 2% BSA and 0.05% Tween-20 (Sigma)) for 1 h at room temperature. Immediately after blocking, monoclonal antibodies or plasma samples were added in PBS and incubated for 1 h at room temperature. Plasma samples were assayed at a 1:66 starting dilution and 7 (IgA and IgM anti-RBD) or 11 (IgG anti-RBD) additional threefold serial dilutions. Monoclonal antibodies were tested at 10 μg ml^−1^ starting concentration and 10 additional fourfold serial dilutions. Plates were washed 6 times with washing buffer and then incubated with anti-human IgG, IgM or IgA secondary antibody conjugated to horseradish peroxidase (HRP) (Jackson Immuno Research 109-036-088 109-035-129 and Sigma A0295) in blocking buffer at a 1:5,000 dilution (IgM and IgG) or 1:3,000 dilution (IgA). Plates were developed by addition of the HRP substrate, TMB (ThermoFisher) for 10 min (plasma samples) or 4 min (monoclonal antibodies). The developing reaction was stopped by adding 50 μl 1 M H_2_SO_4_ and absorbance was measured at 450 nm with an ELISA microplate reader (FluoStar Omega 5.11, BMG Labtech) with Omega MARS software for analysis. For plasma samples, a positive control (plasma from participant COV72, diluted 66.6-fold and seven additional threefold serial dilutions in PBS) was added to every assay plate for validation. The average of its signal was used for normalization of all of the other values on the same plate with Excel software before calculating the area under the curve using Prism v.9.1(GraphPad). For monoclonal antibodies, the EC_50_ was determined using four-parameter nonlinear regression (GraphPad Prism v.9.1).

### Proteins

Mammalian expression vectors encoding the RBDs of SARS-CoV-2 (GenBank MN985325.1; S protein residues 319-539) or K417N, E484K, N501Y RBD mutants with an N-terminal human IL-2 or Mu phosphatase signal peptide were previously described^46^. SARS-CoV-2 nucleocapsid protein (N) was purchased from Sino Biological (40588-V08B).

### SARS-CoV-2 pseudotyped reporter virus

A panel of plasmids expressing RBD-mutant SARS-CoV-2 S proteins in the context of pSARS-CoV-2-S_Δ19_ has previously been described^2,9,26^. Variant pseudoviruses resembling variants of concern B.1.1.7 (first isolated in the UK), B.1.351 (first isolated in South Africa), B.1.526 (first isolated in New York City) and P.1 (first isolated in Brazil) were generated by introduction of substitutions using synthetic gene fragments (IDT) or overlap extension PCR mediated mutagenesis and Gibson assembly. Specifically, the variant-specific deletions and substitutions introduced were: B.1.1.7: ΔH69/V70, ΔY144, N501Y, A570D, D614G, P681H, T761I, S982A, D1118H; B.1.351: D80A, D215G, L242H, R246I, K417N, E484K, N501Y, D614G, A701V; B.1.526: L5F, T95I, D253G, E484K, D614G, A701V; P.1: L18F, R20N, P26S, D138Y, R190S, K417T, E484K, N501Y, D614G, H655Y.

The E484K and K417N/E484K/N501Y (KEN) substitution, as well as the deletions and substitutions corresponding to variants of concern were incorporated into an S protein that also includes the R683G substitution, which disrupts the furin cleaveage site and increases particle infectivity. Neutralizing activity against mutant pseudoviruses were compared to a wild-type SARS-CoV-2 S sequence (NC_045512), carrying R683G where appropriate.

SARS-CoV-2 pseudotyped particles were generated as previously described^3,13^. In brief, 293T cells were transfected with pNL4-3ΔEnv-nanoluc and pSARS-CoV-2-S_Δ19_, particles were collected 48 h after transduction, filtered and stored at −80 °C.

### Microneutralization assay with authentic SARS-CoV-2

Microneutralization assays of SARS-CoV-2 virus were performed as previously described^3^. The day before infection, Vero E6 cells were seeded at 1 × 10^4^ cells per well into 96-well plates. The diluted plasma and antibodies were mixed with SARS-CoV-2 WA1/2020 or the B.1.351 variant and incubated for 1 h at 37 °C. The antibody–virus mix was then directly applied to Vero E6 cells and incubated for 22 h at 37 °C. Cells were subsequently fixed by adding an equal volume of 70% formaldehyde to the wells, followed by permeabilization with 1% Triton X-100 for 10 min. After washing, cells were incubated for 1 h at 37 °C with blocking solution of 5% goat serum in PBS (catalogue no. 005–000-121; Jackson ImmunoResearch). A rabbit polyclonal anti-SARS-CoV-2 nucleocapsid antibody (catalogue no. GTX135357; GeneTex) was added to the cells at 1:1,000 dilution in blocking solution and incubated at 4 °C overnight. Goat anti-rabbit AlexaFluor 594 (catalogue no. A-11012; Life Technologies) was used as a secondary antibody at a dilution of 1:2,000. Nuclei were stained with Hoechst 33342 (catalogue no. 62249; Thermo Fisher Scientific) at 1 μg ml^−1^. Images were acquired with a fluorescence microscope and analysed using ImageXpress Micro XLS (Molecular Devices). All experiments were performed in a biosafety level 3 laboratory.

### Pseudotyped virus neutralization assay

Fourfold serially diluted plasma from COVID-19-convalescent individuals or monoclonal antibodies were incubated with SARS-CoV-2 pseudotyped virus for 1 h at 37 °C. The mixture was subsequently incubated with 293T_Ace2_ cells^3^ (for comparisons of plasma or monoclonal antibodies from convalescent individuals) or HT1080Ace2 cl14 cells^13^ (for analyses involving mutant or variant pseudovirus panels), as indicated, for 48 h after which cells were washed with PBS and lysed with Luciferase Cell Culture Lysis 5× reagent (Promega). Nanoluc luciferase activity in lysates was measured using the Nano-Glo Luciferase Assay System (Promega) with the Glomax Navigator (Promega). The obtained relative luminescence units were normalized to those derived from cells infected with SARS-CoV-2 pseudotyped virus in the absence of plasma or monoclonal antibodies. The NT_50_ or half-maximal and 90% inhibitory concentrations for monoclonal antibodies (IC_50_ and IC_90_) were determined using four-parameter nonlinear regression (least squares regression method without weighting; constraints: top, 1; bottom, 0) (GraphPad Prism).

### Biotinylation of viral protein for use in flow cytometry

Purified and Avi-tagged SARS-CoV-2 RBD or SARS-CoV-2 RBD KEN mutant (K417N, E484K, N501Y) was biotinylated using the Biotin–Protein Ligase–BIRA kit according to manufacturer’s instructions (Avidity) as previously described^3^. Ovalbumin (Sigma, A5503-1G) was biotinylated using the EZ-Link Sulfo-NHS-LC-Biotinylation kit according to the manufacturer’s instructions (Thermo Scientific). Biotinylated ovalbumin was conjugated to streptavidin-BV711 (BD biosciences, 563262) and RBD to streptavidin-PE (BD Biosciences, 554061) and streptavidin-AF647 (Biolegend, 405237)^3^.

### Flow cytometry and single-cell sorting

Single-cell sorting by flow cytometry has previously been described^3^. In brief, peripheral blood mononuclear cells were enriched for B cells by negative selection using a pan-B-cell isolation kit according to the manufacturer’s instructions (Miltenyi Biotec, 130-101-638). The enriched B cells were incubated in FACS buffer (1× PBS, 2% FCS, 1 mM EDTA) with the following anti-human antibodies (all at 1:200 dilution): anti-CD20-PECy7 (BD Biosciences, 335793), anti-CD3-APC-eFluro 780 (Invitrogen, 47-0037-41), anti-CD8-APC-eFluor 780 (Invitrogen, 47-0086-42), anti-CD16-APC-eFluor 780 (Invitrogen, 47-0168-41), anti-CD14-APC-eFluor 780 (Invitrogen, 47-0149-42), as well as Zombie NIR (BioLegend, 423105) and fluorophore-labelled RBD and ovalbumin (Ova) for 30 min on ice. Single CD3^−^CD8^−^CD14^−^CD16^−^CD20^+^Ova^−^RBD^−^PE^+^RBD^−^AF647^+^ B cells were sorted into individual wells of 96-well plates containing 4 μl of lysis buffer (0.5 × PBS, 10 mM DTT, 3,000 units per ml RNasin Ribonuclease Inhibitors (Promega, N2615) per well using a FACS Aria III and FACSDiva software (Becton Dickinson) for acquisition and FlowJo for analysis. The sorted cells were frozen on dry ice, and then stored at −80 °C or immediately used for subsequent RNA reverse transcription. For B cell phenotype analysis, in addition to above antibodies, B cells were also stained with following anti-human antibodies: anti- IgG-PECF594 (BD biosciences, 562538), anti-IgM-AF700 (Biolegend, 314538), anti-IgA-Viogreen (Miltenyi Biotec, 130-113-481).

### Antibody sequencing, cloning and expression

Antibodies were identified and sequenced as previously described^3^. In brief, RNA from single cells was reverse transcribed (SuperScript III Reverse Transcriptase, Invitrogen, 18080-044) and the cDNA stored at −20 °C or used for subsequent amplification of the variable IGH, IGL and IGK genes by nested PCR and Sanger sequencing. Sequence analysis was performed using MacVector. Amplicons from the first PCR reaction were used as templates for sequence- and ligation-independent cloning into antibody expression vectors. Recombinant monoclonal antibodies were produced and purified as previously described^3^.

### Biolayer interferometry

BLI assays were performed as previously described^3^. In brief, we used the Octet Red instrument (ForteBio) at 30 °C with shaking at 1,000 r.p.m. Epitope-binding assays were performed with protein A biosensor (ForteBio 18-5010), following the manufacturer’s protocol ‘classical sandwich assay’. (1) Sensor check: sensors immersed 30 s in buffer alone (kinetics buffer 10x (ForteBio 18-1105) diluted 1x in PBS1x). (2) Capture first antibody: sensors immersed 10 min with Ab1 at 30 μg ml^−1^. (3) Baseline: sensors immersed 30 s in buffer alone. (4) Blocking: sensors immersed 5 min with IgG isotype control at 50 μg ml^−1^. (6) Antigen association: sensors immersed 5 min with RBD at 100 μg ml^−1^. (7) Baseline: sensors immersed 30 s in buffer alone. (8) Association Ab2: sensors immersed 5 min with Ab2 at 30 μg ml^−1^. Curve fitting was performed using the Fortebio Octet Data analysis software (ForteBio). Affinity measurement of anti-SARS-CoV-2 IgGs binding were corrected by subtracting the signal obtained from traces performed with IgGs in the absence of WT RBD. The kinetic analysis using protein A biosensor (ForteBio 18-5010) was performed as follows: (1) baseline: 60 s immersion in buffer. (2) loading: 200 s immersion in a solution with 30 μg ml^−1^ IgGs. (3) baseline: 200 s immersion in buffer. (4) Association: 300 s immersion in solution with wild-type RBD at 200, 100, 50 or 25 μg/ml. (5) dissociation: 600 s immersion in buffer. Curve fitting was performed using a fast 1:1 binding model and data analysis software (ForteBio). Mean *K*_D_ values were determined by averaging all binding curves that matched the theoretical fit with an *R*^2^ value ≥ 0.8.

### Plasma antibody avidity assay

The plasma SARS-CoV-2 antibody avidity assay were performed as previously described^47^.

### Computational analyses of antibody sequences

Antibody sequences were trimmed based on quality and annotated using Igblastn v.1.14. with IMGT domain delineation system. Annotation was performed systematically using Change-O toolkit v.0.4.540^48^. Heavy and light chains derived from the same cell were paired, and clonotypes were assigned based on their V and J genes using in-house R and Perl scripts (Fig. 2d). All scripts and the data used to process antibody sequences are publicly available on GitHub (https://github.com/stratust/igpipeline).

The frequency distributions of human V genes in anti-SARS-CoV-2 antibodies from this study was compared to 131,284,220 IgH and IgL sequences generated by ref. ^49^ and downloaded from cAb-Rep^50^, a database of human shared BCR clonotypes available at https://cab-rep.c2b2.columbia.edu/. On the basis of the 91 distinct V genes that make up the 6,902 analysed sequences from Ig repertoire of the 10 participants present in this study, we selected the IgH and IgL sequences from the database that are partially coded by the same V genes and counted them according to the constant region. The frequencies shown in Extended Data Fig. 4) are relative to the source and isotype analysed. We used the two-sided binomial test to check whether the number of sequences belonging to a specific IgHV or IgLV gene in the repertoire is different according to the frequency of the same IgV gene in the database. Adjusted *P* values were calculated using the false discovery rate (FDR) correction. Significant differences are denoted with stars.

Nucleotide somatic hypermutation and CDR3 length were determined using in-house R and Perl scripts. For somatic hypermutations, IGHV and IGLV nucleotide sequences were aligned against their closest germlines using Igblastn and the number of differences were considered nucleotide mutations. The average mutations for V genes were calculated by dividing the sum of all nucleotide mutations across all participants by the number of sequences used for the analysis.

Immunoglobulins grouped into the same clonal lineage had their respective IgH and IgL sequences merged and subsequently aligned, using TranslatorX v.1.1^51^, with the unmutated ancestral sequence obtained from IMGT/V-QUEST reference directory^52^. GCTree (https://github.com/matsengrp/gctree)^53^ was further used to perform the phylogenetic trees construction. Each node represents a unique IgH and IgL combination and the size of each node is proportional to the number of identical sequences. The numbered nodes represent the unobserved ancestral genotypes between the germline sequence and the sequences on the downstream branch.

### Data presentation

Figures were arranged in Adobe Illustrator 2020.

### Reporting summary

Further information on research design is available in the Nature Research Reporting Summary linked to this paper.

## Online content

Any methods, additional references, Nature Research reporting summaries, source data, extended data, supplementary information, acknowledgements, peer review information; details of author contributions and competing interests; and statements of data and code availability are available at 10.1038/s41586-021-03696-9.

## Supplementary information

Reporting Summary

Supplementary Table 1 Cohort summary.

Supplementary Table 2 Individual participant characteristics.

Supplementary Table 3 Sequences of anti-SARS-CoV-2 RBD IgG antibodies.

Supplementary Table 4 Sequences, half maximal effective concentrations (EC50s) and inhibitory concentrations (IC50s) of the cloned monoclonal antibodies.

Supplementary Table 5 Binding and Neutralization activity of mAbs against mutant SARS-CoV-2 pseudoviruses.

Supplementary Table 6 Neutralization activity of mAbs against mutant SARS-CoV-2 pseudoviruses - Random potently neutralizing antibodies isolated at 1.3 and 12 months.

Supplementary Table 7 Antibody affinities and neutralization - Clonal pairs isolated at 1.3 and 12 months.

Supplementary Table 8 Neutralization activity of mAbs against mutant SARS-CoV-2 pseudoviruses - Clonal pairs isolated at 1.3 and 12 months.

## Data Availability

Data are provided in Supplementary Tables 1–8. The raw sequencing data have been deposited at Github (https://github.com/stratust/igpipeline). This study also uses data from 10.5061/dryad.35ks2 and from 10.1038/s41586-019-0934-8.

## Code availability

Computer code to process the antibody sequences and/or associated with Fig. 2 and Extended Data Fig. 5 is available at GitHub (https://github.com/stratust/igpipeline).
