# Supplementary material for: Naturally enhanced neutralizing breadth against SARS-CoV-2 one year after infection
Source: Nature. 2021 Jun 14;595(7867):426–31. doi: 10.1038/s41586-021-03696-9 (PMC8277577; doi:10.1038/s41586-021-03696-9)
Supplement: Supplementary file 1 — Reporting Summary [file 41586_2021_3696_MOESM1_ESM.pdf]

nature research

Corresponding author(s): Paul D. Bieniasz, Marina Caskey, Theodora Hatziioannou, Michel C. Nussenzweig

Last updated by author(s): Jun 1, 2021

Reporting Summary

Nature Research wishes to improve the reproducibility of the work that we publish. This form provides structure for consistency and transparency in reporting. For further information on Nature Research policies, see our Editorial Policies and the Editorial Policy Checklist.

Statistics

For all statistical analyses, confirm that the following items are present in the figure legend, table legend, main text, or Methods section.

n/a	Confirmed	
☐	☒	The exact sample size (n) for each experimental group/condition, given as a discrete number and unit of measurement
☐	☒	A statement on whether measurements were taken from distinct samples or whether the same sample was measured repeatedly
☐	☒	The statistical test(s) used AND whether they are one- or two-sided<br>*Only common tests should be described solely by name; describe more complex techniques in the Methods section.*
☒	☐	A description of all covariates tested
☒	☐	A description of any assumptions or corrections, such as tests of normality and adjustment for multiple comparisons
☐	☒	A full description of the statistical parameters including central tendency (e.g. means) or other basic estimates (e.g. regression coefficient) AND variation (e.g. standard deviation) or associated estimates of uncertainty (e.g. confidence intervals)
☐	☒	For null hypothesis testing, the test statistic (e.g. F, t, r) with confidence intervals, effect sizes, degrees of freedom and P value noted<br>*Give P values as exact values whenever suitable.*
☒	☐	For Bayesian analysis, information on the choice of priors and Markov chain Monte Carlo settings
☒	☐	For hierarchical and complex designs, identification of the appropriate level for tests and full reporting of outcomes
☒	☐	Estimates of effect sizes (e.g. Cohen's d, Pearson's r), indicating how they were calculated

Our web collection on statistics for biologists contains articles on many of the points above.

Software and code

Policy information about availability of computer code

Data collection	IRIS by iMedRIS version 11.01 for clinical data collection and management; BD FACSDiva Software Version 8.0.2 for flow sorting; Glomax Navigator Promega V.3 for neutralization assays; Omega 5.11 by BMG Labtech was used for Elisa Assays.
Data analysis	FlowJo 10.6.2 for FACS analysis; GraphPad Prism V_9.1; Microsoft Excel 16.36; MacVector 17.5.4 for sequence analysis; Omega MARS V2.10 by BMG Labtech for luminometer; Glomax Navigator V.3 from Promega, Adobe Illustrator 2020, Igblastn v.1.14 and Translator X v.1.1 for antibody sequences analysis; scripts and the data used to process antibody sequences are available on GitHub (https://github.com/stratust/igpipeline), GCTree for phylogenetic trees construction, ImageXpress Micro XLS for Imaging, ForteBio for Octet data analysis.

For manuscripts utilizing custom algorithms or software that are central to the research but not yet described in published literature, software must be made available to editors and reviewers. We strongly encourage code deposition in a community repository (e.g. GitHub). See the Nature Research guidelines for submitting code & software for further information.

Data

Policy information about availability of data

All manuscripts must include a data availability statement. This statement should provide the following information, where applicable:

- Accession codes, unique identifiers, or web links for publicly available datasets
- A list of figures that have associated raw data
- A description of any restrictions on data availability

Data are provided in SI Table 1-8. The raw sequencing data and computer scripts associated with Figure 2 has been deposited at Github (https://github.com/stratust/igpipeline). This study uses mammalian expression vectors encoding the RBDs of SARS-CoV-2 (GenBank MN985325.1), and uses data from “A Public Database of Memory and Naive B-Cell Receptor Sequences” (https://doi.org/10.5061/dryad.35ks2), and from “High frequency of shared clonotypes in human B cell receptor repertoires” (https://doi.org/10.1038/s41586-019-0934-8).

April 2020

Field-specific reporting

Please select the one below that is the best fit for your research. If you are not sure, read the appropriate sections before making your selection.

☒ Life sciences ☐ Behavioural & social sciences ☐ Ecological, evolutionary & environmental sciences

For a reference copy of the document with all sections, see nature.com/documents/nr-reporting-summary-flat.pdf

Life sciences study design

All studies must disclose on these points even when the disclosure is negative.

Sample size	Sample size of 63 individuals was based on how many of our initial (1.3/6.2 months) study participants we were able to recruit for a return visit for blood donation between February 8 to March 26, 2021
Data exclusions	No data were excluded from the analysis.
Replication	All experiments successfully performed at least twice.
Randomization	This is not relevant as this is an observational study.
Blinding	This is not relevant as this is an observational study.

Reporting for specific materials, systems and methods

We require information from authors about some types of materials, experimental systems and methods used in many studies. Here, indicate whether each material, system or method listed is relevant to your study. If you are not sure if a list item applies to your research, read the appropriate section before selecting a response.

Materials & experimental systems

n/a	Involved in the study	
☐	☒	Antibodies
☐	☒	Eukaryotic cell lines
☒	☐	Palaeontology and archaeology
☒	☐	Animals and other organisms
☐	☒	Human research participants
☒	☐	Clinical data
☒	☐	Dual use research of concern

Methods

n/a	Involved in the study	
☒	☐	ChIP-seq
☐	☒	Flow cytometry
☒	☐	MRI-based neuroimaging

Antibodies

Antibodies used

Mouse anti-human CD20-PECy7 (BD Biosciences, 335793), clone L27
Mouse anti-human CD3-APC-eFluro 780 (Invitrogen, 47-0037-41), clone OKT3
Mouse anti-human CD8-APC-421eFluro 780 (Invitrogen, 47-0086-42), clone OKT8
Mouse anti-human CD16-APC-eFluro 780 (Invitrogen, 47-0168-41), clone eBioCB16
Mouse anti-human CD14-APC-eFluro 780 (Invitrogen, 47-0149-4), clone 61D3
Mouse anti-human IgG-PECF594 (BD biosciences, 562538),
Mouse anti-human IgM-AF700 (Biolegend, 314538),
Mouse anti-human IgA-Viogreen (Miltenyi Biotec, 130-113-481).
Zombie NIR (BioLegend, 423105)
Peroxidase Goat anti-Human IgG Jackson Immuno Research 109-036-088
Peroxidase Goat anti-Human IgM Jackson Immuno Research 109-035-129
Peroxidase Goat anti-Human IgA Sigma A0295

Validation

No validation statements for the antibodies that are commercially available.

Eukaryotic cell lines

Policy information about cell lines

Cell line source(s)

293T(CRL-11268) and HT1080(CCL-121) have been obtained from the ATCC. Based one these cell lines, we generated the 293T/ACE2* and HT1080/ACE2.cl14 cells, which are described in (Robbiani, D. et al. Nature 584, doi.org/10.1038/s41586-020-2456-9)and (Schmidt, F. et al. J Exp Med 217, doi:10.1084/jem.20201181).

Authentication	Not authenticated after purchase from ATCC.
Mycoplasma contamination	The cells were checked for mycoplasma contamination by Hoechst staining, and tested negative.
Commonly misidentified lines (See ICLAC register)	No commonly misidentified cell lines were used.

Human research participants

Policy information about studies involving human research participants

Population characteristics	Previously enrolled study participants were asked to return for a 12-month follow-up visit at the Rockefeller University Hospital in New York from February 8 to March 26, 2021. Eligible participants were adults aged 26-73 years and were either diagnosed with SARS-CoV-2 infection by RT-PCR (cases), or were close contacts (e.g., household, co-workers, members of same religious community) with someone who had been diagnosed with SARS-CoV-2 infection by RT-PCR (contacts). We analyzed 36 males and 27 females with an average age of 47 years. Exclusion criteria included presence of symptoms suggestive of active SARS-CoV-2 infection. Participants that presented with persistent symptoms attributable to COVID-19 were identified on the basis of chronic shortness of breath or fatigue, deficit in athletic ability and/or three or more additional long-term symptoms such as persistent unexplained fevers, chest pain, new-onset cardiac sequalae, arthralgias, impairment of concentration/mental acuity, impairment of sense of smell/taste, neuropathy or cutaneous findings as previously described.
Recruitment	Study participants were recruited at the Rockefeller University Hospital in New York between February 8 to March 26, 2021. Most study participants were residents of the Greater New York City tri-state region and were asked to return approximately 12 months after the time of onset of COVID-19 symptoms. Participants presented to the Rockefeller University Hospital for blood sample collection and were asked about potential symptom persistence since their 6.2 month study visit, laboratory-confirmed episodes of reinfection with SARS-CoV-2, and whether they had received any COVID-19 related treatment or SARS-CoV-2 vaccination in the interim. Other than these criteria no other parameters were used to exclude or include patients. Therefore, we cannot identify any factors that would lead to self-selection bias.
Ethics oversight	Institutional Review Board (IRB) at the Rockefeller University, protocol DRO-1006.

Note that full information on the approval of the study protocol must also be provided in the manuscript.

Flow Cytometry

Plots

Confirm that:

- [x] The axis labels state the marker and fluorochrome used (e.g. CD4-FITC).
- [x] The axis scales are clearly visible. Include numbers along axes only for bottom left plot of group (a 'group' is an analysis of identical markers).
- [x] All plots are contour plots with outliers or pseudocolor plots.
- [x] A numerical value for number of cells or percentage (with statistics) is provided.

Methodology

Sample preparation	Whole blood samples were obtained from study participants recruited through Rockefeller University Hospital. Peripheral blood mononuclear cells (PBMCs) were separated by Ficoll gradient centrifugation. Prior to sorting, PBMCs were enriched for B cells using a Miltenyi Biotech pan B cell isolation kit (cat. no. 130-101-638) and LS columns (cat. no. 130-042-401).
Instrument	FACS Aria III (Becton Dickinson)
Software	BD FACSDiva Software Version 8.0.2 and FlowJo 10.6.2
Cell population abundance	Sorting efficiency ranged from 40% to 80%. This is calculated based on the number of IgG-specific antibody sequences that could be PCR-amplified successfully from single sorted cells from each donor.
Gating strategy	Cells were first gated for lymphocytes in FSC-A (x-axis) versus SSC-A (y-axis). We identify single cells in FSC-A versus FSC-H, and then SSC-A versus SSC-W. We then select for CD20+ Dump- B Cells in dump (anti-CD3-eFluro 780, anti-CD16-eFluro 780, anti-CD8-eFluro 780, anti-CD14-eFluro 780, Zombie NIR) versus CD20 (anti-CD20-PE-Cy7); dump-negative was considered to be signal less than 250, and CD20-positive was taken to be signal greater than 100. We then gate for Ova- B cells in FSC-A versus Ova-BV711; Ova-negative was considered to be all cells with signal less than 100. Select for Sars-CoV-2 RBD double-positive cells in RBD PE versus RBD AlexaFluor 647; this gate was made along the 45° diagonal, above 1000 on both axes.

- [x] Tick this box to confirm that a figure exemplifying the gating strategy is provided in the Supplementary Information.
